# Development of a Danish Adapted Healthy Plant-Based Diet Based on the EAT-Lancet Reference Diet

**DOI:** 10.3390/nu12030738

**Published:** 2020-03-11

**Authors:** Anne D. Lassen, Lene M. Christensen, Ellen Trolle

**Affiliations:** Division of Risk Assessment and Nutrition, National Food Institute, Technical University of Denmark, DK-2800 Kgs. Lyngby, Denmark; lmch@food.dtu.dk (L.M.C.); eltr@food.dtu.dk (E.T.)

**Keywords:** healthy and sustainable diet, nutrition, food based dietary guidelines, nutrient density, meat reduction

## Abstract

Plant-based diets have been linked to both health benefits and a lower climate impact. However, plant-based diets may represent both healthy and unhealthy dietary practices. The present study aimed to develop a nationally adapted healthy plant-based diet based on the global EAT-Lancet reference diet. Development took place in a series of steps. First, the original EAT-Lancet reference diet was evaluated based on food availability, i.e., using Danish food data (Model 1). Then, the model was further modified to reflect national food based dietary guidelines (FBDG) and characteristics of current consumption pattern, e.g., by including processed food, discretionary foods and beverages in the diet (Model 2). The contents of macronutrients, vitamins and minerals, except for vitamin D and iodine, were found to be sufficient for Model 2, according to the recommended nutrient density to be used for planning diets for groups of individuals aged 6–65 years. In addition, the study gave an insight into the nutrients and foods to be aware of in planning a predominantly plant-based diet, thereby providing directions for future revisions of sustainable FBDGs. These include a stronger emphasis on the intake of legumes, nuts and seeds, fruit and vegetables including dark green vegetables, whole-grain products and vegetable oils as well as lowering meat intake.

## 1. Introduction

The concept of a sustainable healthy diet is high on the global political agenda [1,2,3]. The Food and Agriculture Organization (FAO) and World Health Organization (WHO) define sustainable healthy diets as “dietary patterns that promote all dimensions of individuals’ health and wellbeing, have low environmental pressure and impact; are accessible, affordable, safe and equitable; and are culturally acceptable [3]”. This is also reflected in the United Nations’ 17 Sustainable Development Goals (SDGs), where one of the goals, SDG2, focuses on eliminating hunger and malnutrition and improving the sustainability of food systems [4,5].

Achieving healthy diets for everyone from sustainable food systems will require major improvements in the efficiency of food production, large reductions in food losses and waste, as well as substantial shifts in dietary patterns [6,7]. The necessary shifts in dietary pattern include moving towards a more plant-based diet [8,9,10]. The term plant-based diet encompasses a wide variety of dietary patterns which contain lower amounts of animal-source foods, such as meat, and higher amounts of plant-source foods [11].

The negative climate impact (greenhouse gas emissions) resulting from more plant-based diets (both theoretic and real plant-based diet scenarios, i.e., self-selected diets) has been found to be ∼20–35% lower than regular diets, and ∼45–50% lower than vegan diets of the currently commonly consumed diets in high-income countries [12,13]. Variable effects on land use have been reported, e.g., a median reduction of ∼15% for diets with meat partially replaced by plant-based food [12] and a median reduction ∼50% for vegan diets [13]. On the other hand, replacing animal-based foods in the diet with plant-based foods does not always imply lower blue water use (surface and groundwater), as e.g., fruits, nuts and pulses can be more dependent on irrigation than animal foods [14]. This may particularly be a problem in areas with declining groundwater or surface water availability [7,14]. Some studies are now using a water scarcity weighted footprint metric for this purpose, however such studies remain relatively rare [14].

Plant-based diets have been further linked to health benefits, i.e., lower risk of cancer [15], type 2 diabetes [16], and cardiovascular diseases [17]. However, plant-based diets may represent a mix of both healthy and unhealthy dietary practices. Studies have found that plant-based dietary patterns, which emphasized less healthy plant foods (e.g., sweetened beverages, refined grain products, fries and sweets) were associated with higher cardiovascular disease and type 2 diabetes risks as well as total mortality [18,19,20]. Baden et al. points out that public health efforts towards lower chronic disease risk should account for the quality of plant foods [20] and Tuomisto et al. advocate that major efforts are needed to educate people in constructing nutritionally adequate plant-based diets to avoid potential unintended health consequences, e.g., micronutrient deficiencies [21].

The Nordic diet (ND), as adopted from the Opus project [22], is an example of a healthy dietary template that includes the high intake of, e.g., fruits, vegetables, nuts and whole grains and contains less meat (including free-range livestock and game)—around two thirds of the amount of meat consumed in the average Danish diet. It was developed in 2004 by leading Nordic chefs, and was based on four core principles: health, gastronomic potential, sustainability and Nordic identity [23]. 

More recently, the EAT-Lancet Commission has proposed a global healthy reference diet that could help limit environmental changes within the planetary boundaries [7]. The EAT-Lancet Commission explains that the scientific targets for the healthy reference diet were based on extensive literature on foods, dietary patterns and health outcomes, but the context for reviewing the literature is unclear. The EAT-Lancet reference diet contains around one third of the amount of meat consumed by Danes with somewhat larger amounts of fish [24]. Wang et al. calculated the preventable premature deaths achievable by shifting from current national diets to the reference diet from the EAT-Lancet Commission, modified slightly to align with available dietary data, and estimated a substantial reduction in premature deaths, ∼25% of total deaths globally [25]. Further, Hirvonen et al. assessed the relative affordability of the EAT–Lancet reference diet by comparing the total cost per day to 159 countries’ national incomes. It was concluded that the reference diet is affordable for most of the world’s people, but not for people in low-income countries [26]. 

Work is needed to adapt the EAT-Lancet reference diet to national preferences and contexts, e.g., food culture as well as local food availability, the nutrient content of foods and national dietary recommendations [7,21,27]. To our knowledge, results on the adaptation of the reference diet to a local context have not yet been published in the literature. National experiences on methods and considerations about increasing or decreasing the amount of certain foods in the diet for adaptation of the EAT-Lancet reference diet could be helpful for researchers from other countries. Recently, Blackstone et al. compared the EAT-Lancet reference diet with the Dietary Guidelines for Americans and found several areas of agreement between the EAT-Lancet reference diet and the Dietary Guidelines for Americans, but key differences on the amounts of whole grains, fruits, starchy vegetables, red meat, nuts and seeds, and discretionary calories [28].

Denmark has two sets of official dietary recommendations: the Nordic Nutrition Recommendations (the NNR), which provide a basis for evaluating the intake of nutrients and planning diets [29], and the Danish food-based dietary guidelines (FBDG) [30], which provide advice on foods and food groups which supply the required nutrients and promote overall health and prevent chronic diseases [31]. The NNR has been revised and updated five times over the last forty years, most recently in 2012, with a sixth update planned for 2022 [32]. Environmental sustainability is discussed in one chapter in the current NNR, however, sustainability has not been integrated into the Danish FBDG. So far, the sustainability of diets has only been incorporated into relatively few FBDG, e.g., in Sweden [33], Germany [34], UK [35] and the Netherlands [36]. FAO points out that countries should begin a process of incorporating sustainability into national dietary guidelines. To have a real effect on the environmental impact of diets, they need, among other aspects, to be accessible but ambitious, i.e., consider current consumption patterns and the cultural context, but at the same time promote a clear change in the consumption patterns to foster truly sustainable dietary patterns [37].

The present study aimed to develop a nutritionally adequate and culturally adapted plant-based diet based on the EAT-Lancet reference diet. Firstly, the original EAT-Lancet reference diet was evaluated using Danish food data (Model 1). Secondly, modification of the diet was made in order to be consistent with the Danish Dietary Recommendations, taking into account characteristics of current food consumption patterns (e.g., intake of processed foods) and food availability (e.g., only few fortified products), as well as ensuring the nutritional quality of the diet according to the NNR (Model 2). Based on this, we discuss points to consider when moving towards a more healthy and sustainable plant-based diet and revising the Danish FBDG to incorporate environmental sustainability.

## 2. Materials and Methods

Two models were developed and nutritionally assessed. Model 1 corresponds to the reference diet as defined by the EAT-Lancet Commission, but with Danish food data and adjusted to a total energy intake level of 10 MJ (Step 1 and Step 2); and Model 2 further modified to be consistent with the Danish FBDG and to a certain degree the Danish food preferences (Step 3 to Step 6). 

**Figure 1:**
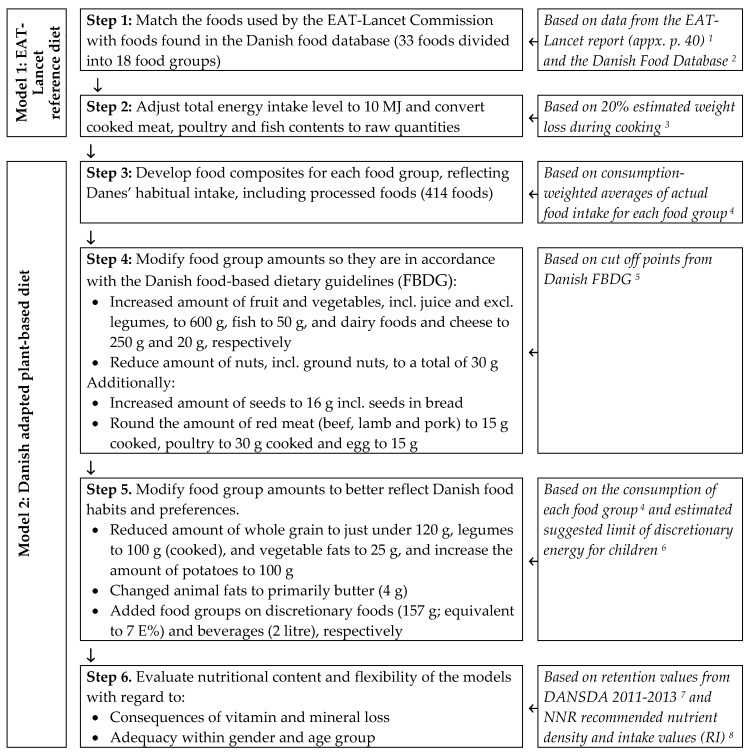
Overview of the steps used in the development of the Danish adapted plant-based diet based on the EAT-Lancet reference diet. ^1^ [7], ^2^ [39], ^3^ [43], ^4^ Extract from The Danish National Survey of Diet and Physical Activity 2011–2013 (DANSDA) (age 15–75 years), ^5^ [30], ^6^ [44], ^7^ [24], ^8^ Age groups 2–5 years, 6–9 years, 10–13 years, 14–17 years, 18–30 years, 31–60 years, 61–74 years [29].

Figure 1 shows an overview of the steps used in the development process. Microsoft Excel spreadsheets were used to compile and calculate the nutritional content of the models. 

### 2.1. Development of the EAT-Lancet Reference Diet with Danish Foods (Model 1)

In Step 1, the 33 foods divided into 18 food groups used in the nutrient calculations performed by the EAT-Lancet Commission as shown in the supplementary appendix (p. 40), were identified [38]. The Danish Food Database was used [39] together with newer analysis not yet incorporated into the database, i.e., the nutritional content of salmon, several cereals, seeds and nuts [40,41]. 

Step 2 included the adjustment of the total energy intake level to 10 MJ to compare with the official Danish FBDG and the NNR. Further, meat, poultry and fish were converted from cooked to raw quantities. The original EAT-Lancet reference diet, the Danish FBDG and the average consumption by food group of Danes aged 15–75 years are shown in Supplementary Material Table S1.

### 2.2. Development of a Danish Adapted Plant-Based Diet (Model 2)

In Step 3, the number and amounts of individual foods within each food group were increased to include foods in the same proportion as consumed in the Danish National Survey of Diet and Physical Activity (DANSDA) 2011–2013, which consists of 7-day food records from a total of 3,189 adults aged 15–75 years [24]. This was done in order to reflect Danes’ habitual intake, including the consumption of processed food (e.g., bread, spreads and discretionary foods). A total of 414 foods were included in the final model. Of these, only a few foods are fortified (salt and salt in bread with iodine, and margarine with vitamin A). 

In Step 4, the amount of foods was modified to be in accordance with the Danish FBDG. On this background, the amount of fruits and vegetables was increased to 600 g per day, including 300 g vegetables, 240 g fruits and 60 g juice (Table 1). In accordance with the EAT-Lancet reference diet, legumes were placed in a separate food group and not included in the vegetable group, as they are in the Danish FBDG. Further, in accordance with the EAT-Lancet reference diet the vegetable group was divided equally into dark green vegetables, red and orange vegetables and other vegetables, based on U.S. Department of Agriculture’s definition in order to meet requirements for e.g., iron and calcium [42]. 

The amount of fish was increased to 50 g per 10 MJ, including 29 g oily fish (cooked) to increase the content of e.g., vitamin D, selenium and n-3 fatty acids in the diet and to be in concordance with the Danish recommended amount of at least 350 g fish and shellfish per week, including 200 g oily fish.

The Danish FBDG states that 250–500 g milk and dairy product daily, as well as 15–25 g cheese per day, are appropriate amounts. On this background, we included 250 g milk and dairy products and 20 g cheese.

Although peanuts are botanically legumes, peanuts/groundnuts are included in the recommendation for nut intake in the Danish FBDG. Up to around 30 g of unsalted nuts and almonds is considered a suitable amount in a healthy diet. On this background we included 15 g peanuts and 15 g nuts and almonds in the model. We also included 16 g seeds including seeds from bread, as seeds were found to increase the content of, e.g., zinc and selenium in the diet. 

Finally, amounts of red meat (beef, lamb and pork) were rounded to 15 g, poultry to 30 g (cooked) and egg to 15 g in order to make it easier to communicate.

In Step 5, food group amounts were modified to be more in accordance with the Danish food habits and preferences. The EAT-Lancet reference diet include whole grains (dry, raw). A high amount of whole grain is associated with a reduced risk of cardiovascular disease and type 2 diabetes as well as certain types of cancer, and in addition helps to ensure a sufficient content of micronutrients [31]. However, refined grain products were included in the model to make it more realistic and to reflect the Danish preferences. Whole grain content requirements for products with the Danish whole grain logo is not 100%. For example, for pasta and noodles, at least 60% whole grain calculated as product dry matter, is required [45]. Therefore, the amount of whole grain was reduced to 116 g (Table 1). The Danish recommendation for whole grain intake is at least 75 g per 10 MJ. The total amount of bread, pasta, rice, cereals and flour/grits were also used to maintain isocaloric content and ended at around 390 g (240 g bread, 30 g pasta (cooked), 24 g rice (cooked), 10 g cereals, 35 g flour/grits (cooked)). A weight change factor of 2.5 was used for rice, pasta, flour and grits [43].

The amount of potatoes included in the model was increased to 100 g. This amount of potatoes was chosen as boiled and baked potatoes are an important part of the Danish food culture, and potatoes contain many vitamins, minerals and dietary fibre. Contrariwise, the amount of legumes was—compared to the EAT-Lancet reference diet—reduced to 40 g in total (100 g cooked), including white and brown beans, dry peas, lentils, chickpeas and soy beans. For comparison, so far Danes’ average intake is only a few grams per day [24]. Legumes contribute, e.g., protein, dietary fibre and micronutrients such as iron, calcium and zinc.

To add versatility to the diet and to ensure a realistic food intake pattern, it is relevant to leave room for discretionary energy, i.e., energy from foods that can be consumed when the essential nutrient needs are fulfilled, including foods with a higher sugar, fat and/or salt content, e.g., chocolate, sweets, cakes, ice cream, desserts, chips and popcorn as well as sugar sweetened drinks and alcohol [44]. The EAT-Lancet reference diet only contains added sugars and not processed food or alcohol. Discretionary foods should be consumed in moderation to make room for more nutrient-dense foods. This also applies to plant-based diets. Biltoft-Jensen et al. suggest a maximum discretionary food content of 10 E% for an energy intake of 10 MJ, and 7 E% for an energy intake of less than 10 MJ [46]. To increase the nutrient density of the model, we included 7 E% discretionary foods, corresponding to 700 kJ in the model. This is about a third of the amount eaten by Danes aged 15–75 years on average. 

Because of the energy and fat content of the discretionary foods and the other processed foods included in Model 2, only 25 g added fat (vegetable based) was included in the model. Further, 4 g butter was included and only a very small amount of lard/tallow, as virtually none of these products are consumed according to the Danish survey. The amount of butter included in the model is significantly lower than the average amount consumed by the adult Danish population (on average 13 g per 10 MJ), to keep the level of fat and saturated fatty acids within the recommendations from NNR.

We added a food group containing tap water, bottled water, coffee and tea in the model. The amount corresponds to the average amount found in the Danish diet (a total of 2 litres). With these changes, all amounts were still within the possible ranges of the EAT-Lancet reference diet and the Danish FBDG (Supplementary Material Table S1).

Step 6 included evaluating the nutritional content and the flexibility of the model. The nutrient content of the two models was compared with the NNR recommended nutrient density (per 10 MJ) to be used for planning diets for groups of individuals 6–65 years of age with a heterogeneous age and sex distribution. The values are adapted to the reference person requiring the highest dietary nutrient density. These recommendations are not intended for pregnant and lactating women or for adult diets with an energy intake of less than 8 MJ per day or above 12 MJ per day, where a lower density of many nutrients might be adequate. In addition, the effect of an estimated loss of vitamins (10%) and minerals (2.5%) was evaluated based on average cooking loss rates from the Danish National Survey of Diet and Physical Activity 2011–2013 [47] combined with an estimated proportion of foods in the model being cooked (50%). For nutrients that were found to be more than about 5% below the recommended nutrient density, the specific age groups, within the age range of 6–65 years, that did not reach the recommended intake (RI) were identified. The model’s flexibility in relation to young children (2–5 years) and older people (65+ years) was identified by scaling the nutrient content to these groups’ reference values for energy intake. The energy value was based on an average physical activity level (PAL value of 1.6 for adults and 1.39–1.73 for children and adolescents [29]).

## 3. Results

### 3.1. Food Group Contents

Table 1 summarizes the amount of foods in the two models. 

Substantially lower values between Model 1 and Model 2 were seen with regard to whole grain, legumes, and vegetable fats (−47%, −44% and −43%, respectively), and substantially higher amounts of potatoes, fruits and berries, dairy foods (cheese converted to milk equivalents [48]) and fish (113%, 59%, 48% and 85%, respectively) were seen when comparing Model 1 and Model 2. All the values in Model 2 were, however, within the possible ranges set by the EAT-Lancet Commission and the Danish FBDG. Besides, two food groups; “coffee, tea and water” and “discretionary foods” were included. 

### 3.2. Macronutrient Contents

Table 2 shows that both Model 1 and Model 2 met the recommended levels of macronutrients, although the content of n-3 fatty acids only just reached the recommendation in Model 1. Compared to Model 1, the percentage of energy from protein increased by 2 percent points and n-3 fatty acids increased by 0.3 percent points in Model 2, partly because Model 2 contains more fish and cheese. Conversely, the percentage of energy from added sugars decreased by 1 percent point, since energy for alcohol was included in Model 2. Finally, the percentage of energy from fat decreased by 5 percent point, mainly due to a smaller amount of vegetable fats and nuts. The content of essential amino acids in the modelled diets is shown in Supplementary Material Table S2.

### 3.3. Vitamin Contents

Table 3 shows that both Model 1 and Model 2 reached the recommended nutrient density for vitamin E, vitamin B1 (Thiamine), vitamin B2 (Riboflavin), niacin, vitamin B6, folate, vitamin B12 and vitamin C. However, vitamin B2 and vitamin B12 only just reached the NNR in Model 1.

The content of vitamin A reached the recommended nutrient density in Model 2, partly because a small amount of liver paste was included, as well as a small amount of margarine, blended spread, and butter, which were included in Model 2 to reflect the Danes’ preferences for fats and spreads. When testing the flexibility of the model, the content of vitamin A was under the RI for men aged 61–74 years. Finally, the vitamin D content was higher in Model 2 compared to Model 1, as the amount of fish was increased in Model 2. It is still well below the recommended nutrient density, and the content does not reach the RI for any groups of the population.

### 3.4. Mineral contents

Table 4 shows that both Model 1 and 2 are well over the recommended nutrient density for potassium, magnesium and phosphorus. In Model 1, the content of calcium, zinc, iodine and selenium is below the NNR.

After adapting the EAT-Lancet reference diet to the Danish FBDG and preferences, Model 2 just met the recommended nutrient density for 6–65 years old with regard to calcium, iron, zinc and selenium. 

The content of sodium was only about 400 mg in Model 1, because salt in processed foods was not addressed in the EAT-Lancet reference diet. In Model 2, salt from processed foods was included, e.g., from bread and cold cuts. Added salt in cooking at home was not included in the model.

Model 2 contained more iodine than model 1, due to the inclusion of iodine from drinking water and enriched salt in bread, as well as a larger amount of fish and dairy. Still, the final model was about 10% under the recommended nutrient density. 

When testing the model’s flexibility, calcium and iron also did not quite reach the recommendation for children aged 2–15 years. The RI of calcium for children aged 2–5 years is 600 mg [29]. With an average energy requirement of 5.3 MJ, the content of calcium ended at about 525 mg. The content of selenium was much higher in Model 2 compared to Model 1, mainly because of almost twice as much fish in Model 2. When testing the flexibility of the model, the content of selenium was under the recommended intake for adults aged 61–74 years. 

## 4. Discussion

The Danish adapted plant-based diet based on the EAT-Lancet reference diet (Model 2) provides an example of a healthy diet with a limited amount of meat and including only few fortified products. All amounts of foods in the adapted diet were within the limits set by the EAT-Lancet reference diet and the Danish FBDG. Additionally, we included 7 E% from discretionary foods and drinks in the diet, as well as beverages such as tap water, bottled water, coffee etc. 

The macronutrient content of the diet as well as the contents of vitamins and minerals, except for vitamin D and iodine, were found to be adequate according to the NNR recommended nutrient density used for planning diets for groups of individuals aged 6–65 years. The high content of legumes and the content of, e.g., fish, balanced out the decrease in protein from meat. Moreover, the low content of some plants in specific amino acids is compensated for by dietary mixtures including legumes and whole-grain products [29,49], resulting in an adequate content of essential amino acids (Supplementary Material Table S2).

The present study also highlights that some vitamins and minerals may need special attention in terms of getting adequate content in the diet. While the selenium content in the original EAT-Lancet reference diet using Danish food composition data (Model 1) was below recommendation, the content was adequate according to the recommended level in Model 2. A low dietary selenium content is a common finding in current diets in Europe as soil concentration of selenium is low compared with other parts of the world [50,51]. Consequently, plant-based foods grown in Europe are relatively low in selenium [50,51], making it difficult to get enough selenium from plant-based sources alone. 

Fish and shellfish are important dietary sources of selenium in Denmark [50]. The Danish adapted plant-based diet included 50 g fish per 10 MJ. In the FBDG, dietary advice emphasizes the intake of fatty fish, due to its high levels of n-3 fatty acids. However, lean fish also contains numerous nutrients, such as protein, vitamin B12, vitamin D, iodine, and selenium [52]. The NNR state that there is a potential conflict between dietary recommendations for fish intake and sustainability considerations [29]. Hallström et al. suggest that seafood that would benefit both health and climate include pelagic species like sprat, herring and mackerel. Seafood with a higher climate impact in relation to their nutritional value (e.g., shrimp, pangasius and plaice) should, on the other hand, not be promoted in dietary advice [53]. To guide consumers in making environmentally responsible seafood choices, WWF has developed a sustainable seafood guide [54]. Also, sustainable seafood certification programs may help inform consumers’ buying decisions.

An adequate amount of vitamin D is known to be challenging to reach, due to a limited amount naturally available in many food products, and the impact of a potential lack of sun exposure, especially in the winter months, is reflected in the vitamin D status [29,55,56]. Unlike many other countries, on the Danish market there are only very few vitamin D fortified foods, but some are seen in categories such as fat spreads, sports drinks and lactose-free milk products [55]. With regards to iodine, low intake is also a common concern. The Danish Veterinary and Food Administration in the year 2000 introduced mandatory fortification with iodine of household salt and salt used as an ingredient in bread and bakery products as a means of controlling iodine deficiency. The level is increased to 20 mg iodine per kg salt from 2019 [57]. Therefore, the intake of iodine in the Danish population is expected to increase and, likewise, the iodine content of the plant-based diets will increase compared to the results found in the present study. 

Model 1 did not reach the NNR recommended density for vitamin A and calcium, and just reached the recommendations for vitamin B2 and vitamin B12, all found to be adequate in Model 2 due to an increase in, e.g., milk and cheese. The Danish adapted plant-based diet included 250 g milk per 10 MJ, which is at the lower end of the suggested range of the Danish FBDG, and slightly lower compared to the Danish average adult diet. Moreover, 20 g cheese per 10 MJ was included to make the diet more in accordance with Danish food culture. With regard to younger children aged 2–5 years having an energy intake around half of 10 MJ, the content of calcium of the diet (Model 2) was found to be below the recommended intake. This indicates a need for a higher content of dairy products or other calcium rich food products to make the diet adequate for all individuals of this age group. Also, higher amounts of dairy products might be needed in case e.g., iodine intake from other food sources, like iodized salt, is low. FAO states that from an environmental point of view there is a need for a better understanding of the role and impact of dairy products in relation to health and sustainability [37].

Additionally, iron and zinc may require special attention in order to obtain adequate amounts from a plant-based diet, especially regarding diets for women of fertile age and young children, which might require a relatively high iron intake. Guidelines should therefore be made to eat plant-based foods rich in iron and zinc, e.g., whole-grain products, legumes, nuts and seeds, and dark-green vegetables rich in iron [21], as well as substances that may facilitate the absorption of iron and zinc, e.g., animal tissue such as meat and fish, as well as vitamin C exerts an enhancing effect on iron absorption [58]. Moreover, food processing practices have the potential to reduce the phytate content of plant-based foods, thus minimizing the adverse effect on mineral bioavailability [59,60]. 

In Model 2, the salt content increased compared to Model 1, and almost reached maximum recommended sodium intake per day, because of the inclusion of processed foods, e.g., bread products. Neither table salt nor salt added during cooking was included. This highlights the need of the food industry to lower the content of salt in processed foods and in food service to make sure that the current high salt content found in the average diet is not “transferred” to a plant-based diet [61,62]. A recent study showed that the addition of herbs and spices is a feasible strategy for achieving a 50% reduction in salt content without compromising hedonic appreciation [63]. Excess dietary sodium has been found to have a major role in the pathogenesis of hypertension, a leading risk factor for premature death in the world, as it has an effect on blood pressure. In contrary, a higher potassium intake is associated with lower blood pressure [61], and an increase in potassium intake is therefore recommended. In Model 2, the potassium content is 20% higher than the average daily consumption among Danish adults (4.5 g pr. 10 MJ in Model 2 vs. 3.7 g potassium pr. 10 MJ among adults). 

In general, climate impact and land use have been found to be highest in the production of ruminant meat (e.g., cows, sheep and goats), followed by other animal products (e.g., pork, poultry, cheese) and lower in the production of most plant-based foods (where rice and some nuts, berries, and vegetable oils are among the plant-based foods with the highest greenhouse gas emissions) [64,65]. Higher emission and land use are found for beef originating from beef herds, compared to beef originating from dairy herds [65]. 

Based on health aspects, the World Cancer Research Fund International and the American Institute for Cancer Research recommend that you eat no more than 350–500 g (cooked weight) red meat, such as beef, pork and lamb per week and little if any, processed meat [66]. Consequently, reducing red and processed meat intake can be seen as a good step both with respect to reducing climate and environmental impacts, and in relation to heath issues. Some authors, however, argue that the conclusions about the health risk of red meat are not supported by robust scientific evidence [67,68]. As an example, the authors refer to a meta-analysis of RCTs showing that eating meat does not lead to a deterioration of cardiovascular risk markers [69]. In an updated meta-analysis of RCTs it is concluded that inconsistencies regarding the effects of red meat on cardiovascular disease risk factors are attributable, in part, to the composition of the diets. Substituting red meat with high-quality plant protein sources, but not e.g., low-quality carbohydrates, leads to favorable changes in blood lipids and lipoproteins [70]. Meta-analyses of prospective studies have reported positive associations between red meat intake and an increased risk of stroke and type 2 diabetes (T2D) [71,72,73], and both red meat and processed red meat have been associated with all-cause mortality [74] and with cardiovascular disease mortality [75]. Others, e.g., Zeraatkar et al., conclude that the magnitude of associations is small and the certainty is low, although same overall results were found in meta-analyses on cohort studies, i.e., reduced intake of unprocessed red meat intake was associated with reduction in risk for cardiovascular mortality, stroke, myocardial infarction (MI), and T2D; and reduced intake of processed meat intake was associated with decrease in risk for all-cause mortality, cardiovascular mortality, stroke, MI, and T2D [76]. Vernooij et al. point out that the high heterogeneity of dietary patterns across studies is a significant limitation [77], and results from cohort studies indicate that substituting red meat with fish, poultry, nuts, legumes, low fat dairy and/or whole grain was associated with a lower risk of mortality [78], and substituting red meat with nuts, low fat dairy and/or whole grain was associated with lower risk of T2D [79]. In order to get the desired health benefit, it is therefore an important message to communicate that some or all of the meat, especially red and processed meat, in the usual dishes should be replaced with other protein-rich products, preferably plant-based products like legumes and nuts.

The present study demonstrated the adequacy of a plant-based diet where total meat consumption was lowered to around 350 g per week provided the consumption of, e.g., legumes are increased. Different strategies could be adopted to lower meat intake, i.e., through reduced portion sizes and a reduced frequency of intake and instead encourage the replacement of meat by plant-based protein-rich products (e.g., meat-free meals/days). Both encouraging the replacement of meat by plant protein and encouraging a downsizing of portion size have been found to be effective in reducing meat purchases or consumption during experimental studies [80].

In the FBDG, it may be time to provide specific guidelines for the consumption of legumes [81] directed at both the general population and population groups with a predominantly plant-based diet. Legumes should be considered as a separate food group or as part of the protein food group (meat, fish and dairy). Legumes, together with nuts and seeds, are good sources of protein, dietary fibre, various micronutrients and other bioactive components [82]. Nuts and seeds are also high in unsaturated fatty acids [83]. Legumes have been associated with fewer incidences of CVD, CHD, hypertension, and obesity incidence [84] and a newly published systematic review and meta-analysis showed a beneficial role of nut consumption in reducing the incidence of, and mortality from, different CVD outcomes [83]. Röös et al. explored a scenario in which meat consumption in Sweden was reduced by 50% and replaced by domestically grown legumes and found that the climate impact of the average Swedish diet would be reduced by 20% and the land use requirement by 23%, while intake of energy and most macro- and micro-nutrients would comply with NNR [60]. The present study suggests 100 g legumes (cooked weight/10 MJ) as an appropriate average amount in diets low in meat. Moreover, a high amount of nuts (30 g/10 MJ) and seeds (16 g/10 MJ) was included in the Danish adapted plant-based diet compared to the average Danish diet. Current global nut production, however, contributes to, and is affected by, different levels of blue water stress in many regions of the world, and Vanham et al. propose sustainable intensification of nut production using nut-specific water footprint benchmarks [85]. Generally, groundnuts have smaller total and blue water footprints both per kg and per g of protein than tree nuts [85].

Also, a higher amount of vegetables (300 g/10 MJ), especially dark green vegetables, e.g., broccoli, spinach and kale (100 g/10 MJ), was included in the Danish adapted plant-based diet compared to the Danish average diet. Fruit and vegetables and whole-grain products are recommended both in plant-based and omnivore diets. The present study highlights the benefits of each type of vegetables, including both dark green and red/orange vegetables, in order to get adequate nutrients, especially in a plant-based diet. Fungi (mushrooms) in Denmark are not included in the fruit and vegetable recommendation. Due to their aroma properties (e.g., rich in umami substances), mushrooms may help people move toward healthier, plant-based choices [86,87]. 

In the present study, about 120 g whole grain was included in the adapted plant-based diet. The Global Burden of Disease Study estimated the optimal level of intake of whole grain to be at the same level, i.e., 125 g (100–150 g) whole grain per day. Additionally, 100 g potatoes were included in the diet. In a systematic review, Schwingshackl et al. concluded that potato consumption in general is not related to risk of many chronic diseases, but could pose a small increase in the risk of type 2 diabetes if consumed boiled. A clear relation was found between the consumption of French fries and the risk of type 2 diabetes and hypertension [88]. From a sustainability view, potatoes have a very low climate impact [65]. Rice, on the other hand, has high greenhouse gas emissions compared with other plant foods, as methane is emitted when flooding rice fields [64].

In a British study, the environmental impacts of 56 vegetable products were evaluated. For asparagus, transportation was found to be the largest contributor to greenhouse gas emissions, due to a big amount of imported asparagus being airfreighted, and asparagus was found to have the highest per-kg impacts across most of the 19 impact categories considered. Cabbage, celery and Brussels sprouts, on the other hand, were found to be, in general, the most environmentally sustainable [89]. Röös and Karlsson conclude that the reduction in greenhouse gas emissions in relative terms of the individual vegetables might be up to e.g., 60% when prioritizing seasonal produce; however, the reduction in greenhouse gas emissions in absolute terms from eating seasonal vegetables is limited, as emissions from vegetable production make up a minor proportion of the total emissions from food consumption [90]. 

An Australian study has shown that discretionary foods accounts for a significant part of the overall diet-related life cycle water use, greenhouse gas emissions and land use [91]. The present study also highlights the need to avoid excessive consumption of discretionary foods in order to meet nutrient and food group recommendations without exceeding calorie needs. According to the results in a review by Hallström et al., balancing energy intake and expenditure can reduce the climate impact of the diet by 0%–10%, depending on the assumed energy requirements [92]. 

In their guiding principles for sustainable healthy diets, FAO and WHO state that diets should be based on a great variety of unprocessed or minimally processed foods, balanced across food groups, while restricting highly processed food and drink products [3]. Whereas food processing can be beneficial in making food more available as well as safer, some forms of processing can lead to very high densities of salt, added sugars and saturated fats, and these products, when consumed in high amounts, can undermine diet quality [3]. Likewise, Hu et al. suggest that plant-based meat alternatives may have some role in improving human and planetary health, but there is no evidence to suggest that they can substitute for healthy diets focused on minimally processed plant foods such as legumes, nuts and seeds [93].

Table 5 summarizes the main points to consider when adopting a more sustainable heathy plant-based diet. Adequate nutrient content is ensured by eating plenty of fruits and vegetables, including dark green vegetables to provide, e.g., iron and calcium, red/orange vegetables to provide, e.g., vitamin A, legumes, nuts and seeds to provide, e.g., protein, iron, calcium, zinc and selenium, whole-grain products and potatoes to provide, e.g., protein, iron and zinc, and, in addition, moderate amounts of fish to provide e.g., n-3 fatty acids, vitamin D and selenium, milk and dairy products to provide, e.g., B-group vitamins and calcium, vegetable fat/oils to provide e.g., vitamin E and n-3 fatty acids, and poultry and egg also to provide, e.g., B-group vitamins, iron and protein. Additionally, mushrooms could be added because of e.g., their aroma properties. Furthermore, the intake of red and processed meat, discretionary foods, salt and possible ultra-processed foods should be limited or included to a lesser extent. To optimize the diet and ensure a healthy sustainable diet, food waste needs to be minimized and over-consumption avoided. Household routines such as planning, shopping, storing, cooking, eating, and managing leftovers play a decisive role in food provisioning but also in food waste generation [94]. 

A limitation of the study is that the adapted plant-based diet does not encompass small children aged 2–5 years, elderly aged 65+ and pregnant and lactating women, and further that the diet has not been tested in real life. However, a strength of the study is that the model is based on data from the Danish National Survey of Dietary Habits and Physical Activity, which takes into account the food preferences of adults aged 15–75 years and uses data on nutritional content of Danish foods. Fortified foods were not included in the calculations besides salt fortified with iodine and margarine fortified with vitamin A. Another strength of the model is that it turns the amount of foods, i.e., legumes and whole grain, into amounts that are more realistic in a Danish context, although still well above the average intake, i.e., 100 g cooked legumes compared to 178 g in the EAT-Lancet reference diet. It is assumed that the adapted diet in the present study would be applicable in many other countries with the same overall dietary pattern, e.g., Western or Northern European dietary patterns. A limitation that other preferences, e.g., consuming less legumes, increasing the amount of discretionary foods or animal fats or consuming a strictly vegetarian diet, are not tested. This should be further explored. Nevertheless, using the nutrient density approach ensures that the micronutrient requirement of the “most demanding subject” is met, making the model fairly robust and useful for heterogeneous groups of people. 

In order to achieve the desired transformation towards a sustainable healthy diet major and multi-sectorial efforts are needed as well as a strong political commitment [95]. In societies which traditionally have a high consumption of livestock products, this includes educating people in the preparation and composition of nutritionally adequate plant-based diets [21] and in addition balancing food intake with physical activity and minimizing food waste. Schanes et al. conclude that there is also a need to go beyond putting the responsibility solely on individuals [94]. Cooperation with stakeholders along the supply chain are of utmost importance for a more sustainable handling of food [94]. Additionally, there is a need for industry to meet new demands for minimally processed foods, and to minimize the environmental impact in all parts of the food system. Many other changes would be needed including incorporating regulatory, fiscal and voluntary initiatives, to make healthy sustainable foods available and accessible to all segments of the population (e.g., more convenient, affordable and tasty foods) [27]. Darmon et al. found that nutrient-dense foods often cost more [96], so that changing dietary behaviors into a more healthy sustainable direction may also require some economic interventions [96]. Consumers should be guided and nudged towards making healthier and more sustainable food choices at the point of purchase [96,97]. Research is needed to guide these changes at all levels.

Future research should also create more knowledge on how to help change the populations’ dietary habits to a more sustainable diet, taking into account nutritional needs of the specific target groups and different preferences and dietary scenario, including preferences for specific kind of foods, e.g., plant-based dairy alternatives. Moreover, findings from the present study indicate that more research is needed about optimal intake of e.g., legumes, nuts, seeds and mushrooms in plant-based diets, in terms of both possible health benefits and risks as well as environmental impacts. Also, a better understanding of the environmental, health and economic impacts of consuming ultra-processed plant-based food products, e.g., highly processed plant-based meat alternatives, is warranted. In addition, there is a need for more real-life intervention studies which focus on promoting and evaluating health benefits of consuming plant-based foods besides fruits and vegetables [97]. Finally, there is a need to closely follow and assess the impact of the development in the food system to continuously adjust sustainable FBDGs.

## 5. Conclusions

The present study shows that the Danish adapted plant-based diet, consistent with the Danish FBDG, taking into account current food culture and local food availability (e.g., including only few fortified products) is nutritionally adequate within the age range of 6–65 years, except when it comes to vitamin D and iodine, which also are known challenges in the average Danish diet. In addition, the study provides knowledge about nutrients and foods to be aware of when limiting the amount of animal foods, thereby providing directions for future development of sustainable FBDGs. This includes moving toward a diet that has a stronger emphasis on the intake of legumes, nuts and seeds, fruit and vegetables including dark green and red and orange vegetables, whole-grain products, vegetable oils as well as limiting intake of red and processed meat and lowering total meat intake. 

## Figures and Tables

**Table 1:** Content of foods in the EAT-Lancet reference diet with Danish foods (Model 1) and in the Danish adapted plant-based diet (Model 2) per 10 MJ.

Food Groups	Model 1: EAT-Lancet Reference Diet with Danish Foods (g per 10 MJ) ^4^	Model 2: Danish Adapted Plant-Based Diet (g per 10 MJ)
Bread and cereals	220 (dry, raw)	390 (cooked)
Whole grain content	220	116
Potatoes	47	100
All vegetables (excl. legumes)	285	300
Dark green vegetables	95	100
Red and orange vegetables	95	100
Other vegetables	95	100
All fruits and berries, incl. juice	189	300
Dairy foods	236	250
Cheese	Included in dairy foods	20
Beef, lamb, pork, cooked ^1^	12	15
Poultry, cooked ^1^	27	30
Eggs	12	15
Fish, cooked ^1^	27	50
Legumes, cooked ^2^	178	100
Nuts, ground nuts and seeds	48	46
Vegetable fats	44	25
Animal fats	4	4
Coffee, tea and water	Not specified	2000
Discretionary foods ^3^	Not specified	157

^1^ Cooking shrinkage: 20% [43], ^2^ Weight change factor: 2.5 [43], ^3^ Includes sugar sweetened beverages, confectionary, cakes, ice cream, snacks, alcohol etc., ^4^ Values are slightly lower than the original EAT-Lancet reference diet because of the adjustment of the total energy intake level to 10 MJ.

**Table 2:** Content of macronutrients in the EAT-Lancet reference diet with Danish foods (Model 1) and in the Danish adapted plant-based diet (Model 2) per 10 MJ, compared to recommended intake ranges from the Nordic Nutrition Recommendations.

Macronutrients	Model 1: EAT-Lancet Reference Diet with Danish Foods (per 10 MJ)	Model 2: Danish Adapted Plant-Based Diet (per 10 MJ)	Nordic Nutrition Recommendations 2012 ^3^
Protein, g	82	92	
Carbohydrates, g	264	275	
Added, refined sugars, g	29	23	
Fat, g	102	89	
Saturated fatty acids, g	23	24	
n-3 fatty acids, g	3	4	
Alcohol, g	0	5	
Protein, E% ^1^	14	16	10-20 (15)
Carbohydrates, E% ^1^	48	51	45-60 (52-53)
Added, refined sugars, E% ^1^	5.0	4.0	≤10
Dietary fibre, g/10 MJ	43	43	≥30
Fat, E% ^1^	38	33	25-40 (32-33)
Saturated fatty acids, E% ^1^	8.6	8.8	≤10
n-3 fatty acids, E% ^1^	1.0	1.3	≥1
Alcohol, E%	0	1.4 ^2^	<5

^1^ Excl. alcohol, ^2^ Alcohol is exchangeable with added sugars, ^3^ Recommended intake ranges (suitable target for planning purposes) [29].

**Table 3:** Content of vitamins in the EAT-Lancet reference diet with Danish foods (Model 1) and in the Danish adapted plant-based diet (Model 2) per 10 MJ compared to the Nordic Nutrition Recommendations on nutrient density.

Vitamins	Model 1: EAT-Lancet Reference Diet with Danish Foods (per 10 MJ) ^1^	Model 2: Danish Adapted Plant-Based Diet (per 10 MJ) ^1^	Nordic Nutrition Recommendations 2012 ^2^
Vitamin A, RE	751	941	800
Vitamin D, µg	2.5	4.7	14
Vitamin E, α-TE	21	15	9
Vitamin B1, mg	1.9	1.7	1.2
Vitamin B2, mg	1.5	1.8	1.4
Niacin, NE	30	37	16
Vitamin B6, mg	2.5	2.2	1.3
Folate, µg	672	694	450
Vitamin B12, µg	2.4	5.4	2
Vitamin C, mg	153	221	80

^1^ Vitamin loss due to cooking is not subtracted. ^2^ Recommended nutrient density (per 10 MJ) to be used for planning diets for groups of individuals 6–65 years of age with a heterogeneous age and sex distribution [29].

**Table 4:** Content of minerals in the EAT-Lancet reference diet with Danish foods (Model 1) and in the Danish adapted plant-based diet (Model 2) per 10 MJ, compared to the Nordic Nutrition Recommendations on nutrient density.

Minerals	Model 1: EAT-Lancet Reference Diet with Danish Foods (per 10 MJ) ^1^	Model 2: Danish Adapted Plant-Based Diet (per 10 MJ) ^1^	Nordic Nutrition Recommendations 2012 ^2^
Sodium, mg	383	2355	≤2400 ^3^
Potassium, g	3.9	4.6	3.5
Calcium, mg	684	1037	1000
Magnesium, mg	584	555	320
Phosphorus, mg	1787	1769	800
Iron, mg	18	16	16
Zinc, mg	11	12	12
Iodine, µg	85	154	170
Selenium, µg	35	57	57

^1^ Mineral loss due to cooking is not subtracted. ^2^ Recommended nutrient density (per 10 MJ) to be used for planning diets for groups of individuals 6–65 years of age with a heterogeneous age and sex distribution [29], ^3^ Recommended population goal [29].

**Table 5:** Main points to consider when adopting a more sustainable plant-based diet.

Eat More of	Eat Adequate/ Moderate Amounts of	Limit Intake of
Vegetables, including dark green and red/orange vegetables	Fish and seafood (choose the most sustainable)	Red meat (particularly beef) and processed meat
Legumes	Milk (low-fat) and dairy products	Discretionary foods
Nuts and seeds	Vegetable oils	Salt
Fruits and berries	Poultry and eggs	Alcohol
Whole-grain products and potatoes	(Mushrooms)	Ultra-processed foods
Avoid over-consumption and minimize food waste		

## Supplementary Materials

The following are available online at https://www.mdpi.com/2072-6643/12/3/738/s1, Table S1. EAT-Lancet reference diet compared with Danish FBDG /Nordic Nutrition Recommendations (NNR) and Danes average consumption (15–75 years), Table S2. Content of essential amino acids in the EAT-Lancet reference diet with Danish foods (Model 1) and in the Danish adapted plant-based diet (Model 2) per 10 MJ compared to the recommendations from WHO/FAO/UNU.
