# Supplementary material for: Development of a Danish Adapted Healthy Plant-Based Diet Based on the EAT-Lancet Reference Diet
Source: Nutrients. 2020 Mar 11;12(3):738. doi: 10.3390/nu12030738 (PMC7146415; doi:10.3390/nu12030738)
Supplement: Supplementary file 1 [file nutrients-12-00738-s001.pdf]

Supplementary Materials: Development of a Danish adapted healthy plant-based diet based on the EAT-Lancet reference diet

Anne D. Lassen, Lene M. Christensen, and Ellen Trolle

Table S1. EAT-Lancet reference diet compared with Danish FBDG /Nordic Nutrition Recommendations (NNR) and Danes average consumption (15-75 years)

**Food groups**	**EAT-Lancet reference diet (possible ranges)** [1]	**Danish FBDG** [2]**/ (NNR)** [3]	**Average consumption (15-75 years)** [4]
Whole grains, dry, raw	232 g	At least 75 g	58 g
Carbohydrates	(0-60% of energy)	(45-60% of energy)	47% of energy
Potatoes	50 g (0-100 g)	Belongs in a healthy diet	90 g
Vegetables	300 g (200-600 g), excl. legumes	At least 600 g, incl. legumes, incl. fruit juice	220 g
Fruits and berries	200 g (100 – 300 g)	About half vegetables	190 g
Milk	250 g (0-500 g) [5]	250-500 g is appropriate Preferably lean	319 g
Cheese		If a healthy diet 15 g full fat or 25 g lean	45 g
Beef and lamb	7 g (0-14 g) [6]	Not more than 71 g in average Preferably lean	50 g [10]
Pork	7 g (0-14 g) [6]		87 g [10]
Poultry	29 g (0-58 g) [7]		28 g [10]
Eggs	13 g (0-25 g)		23 g
Fish	28 g (0-100 g) [8]	50 g in average About 28 g fatty fish	38 g [10]
Legumes, dry, raw	50 g (0-100 g) [9]	Included in vegetables	1 g
Soy foods	25 g (0-50 g) [9]		2 g
Peanuts	25 g (0-75 g) [9]	Not more than 30 g/day	4 g
Tree nuts	25 g [9]		2 g
Palm oil	6.8 g (0-6.8 g)	Eat less saturated fat Choose plant oils, liquid and soft margarine	12 g [11]
Unsaturated oils	40 g (20-80 g)		11 g [12]
Dairy fats	0 g Included in milk		13 g [13]
Lard or tallow	5 g (0-5 g)		<1 g
Added sugars	31 g (0-31 g)	Eat less sugar (Below 10% of energy)	51 g

[1] For an intake of 2500 kcal equivalent to 10,45 MJ/day [1], [2] For an intake of 10 MJ/day [2], [3] [3], [4] For an intake of 10 MJ/day, n=3189, extract from The Danish National Survey of Diet and Physical Activity 2011-2013, [5] 250 g milk equals 250 milk equivalents [4], [6] Cooked. Beef and lamb are exchangeable with pork and vice versa, [7] Cooked. Poultry is exchangeable with eggs, fish, or plant protein sources, [8] Cooked. [9] Legumes, peanuts, tree nuts, seeds, and soy are interchangeable, [10] Mix of raw and cooked, [11] Vegetable fat with a high content of saturated fatty acids (hard margarine with 20-30% saturated fatty acids, coconut fat, palm oil), [12] Vegetable fat with a low content of saturated fatty acids (margarine with 5-10% saturated fatty acids, mayonnaise, unsaturated oils), [13] Butter and blended spread.

Table S2. Content of essential amino acids in the EAT-Lancet reference diet with Danish foods (Model 1) and in the Danish adapted plant-based diet (Model 2) per 10 MJ compared to the recommendations from WHO/FAO/UNU

Essential amino acids	Model 1: EAT-Lancet reference diet with Danish foods (per 10 MJ)	Model 2: Danish adapted plant-based diet (per 10 MJ)	WHO/FAO/UNU [1]
In total (g/100 g protein)	38	38	27
Isoleucine	4,5	4,5	3,0
Leucine	7,7	7,5	5,9
Lysine	6,2	6,0	4,5
Methionine	1,9	2,0	1,6
Phenylalanine	5,0	4,7	3,8
Threonine	3,8	3,7	2,3
Tryptophan	1,2	1,2	0,6
Valine	5,6	5,6	3,9
Histidine	2,6	2,6	1,5

[1] [5]

Reference List

1. Willett, W.; Rockström, J.; Loken, B.; Springmann, M.; Lang, T.; Vermeulen, S.; Garnett, T.; Tilman, D.; DeClerck, F.; Wood, A.; Jonell, M. Food in the Anthropocene: The EAT-Lancet Commission on healthy diets from sustainable food systems. *Lancet* **2019**, *393*, 447-492.

2. Danish Veterinary and Food Administration: The official dietary guidelines. Available online: https://altomkost.dk/english/#c41067 (accessed on 1. April 2019). Glostrup, Ministry of food, agriculture and fisheries. Danish Veterinary and food Administration, **2013**.

3. Nordic Council of Ministers: Nordic Nutrition Recommendations 2012. Integrating Nutrition and Physical Activity, 5th edn. Nord 2014:002. Copenhagen, **2014**.

4. USDA ChooseMyPlate: What are included in the Dairy group? Available online: https://www.choosemyplate.gov/eathealthy/dairy november 2019 (accessed on 1. April 2019). Glostrup, **2019**.

5. World Health Organization: Protein and Amino acid Requirements in Human Nutrition. Report of a Joint WHO/FAO/UNU Expert Consultation. World Health Organization Technical Report Series. **2007**.
